# Effect of Attentional Focus Instructions on Motor Learning and Performance of Patients with Central Nervous System and Musculoskeletal Disorders: a Systematic Review

**DOI:** 10.3390/jfmk3030040

**Published:** 2018-07-25

**Authors:** Alessandro Piccoli, Giacomo Rossettini, Simone Cecchetto, Antonello Viceconti, Diego Ristori, Andrea Turolla, Filippo Maselli, Marco Testa

**Affiliations:** 1 Department of Neuroscience, Rehabilitation, Ophthalmology, Genetics, Maternal and Child Health, University of Genova, Campus of Savona, 17100 Savona, Italy; 2 Trento Health Care Authority, 38123 Trento, Italy; 3 Fondazione Ospedale San Camillo IRCCS, 30126 Venezia, Italy

**Keywords:** attentional focus, motor learning, performance, exercise therapy

## Abstract

Exercise is one of the main rehabilitative interventions, commonly used to improve performance and motor learning. During the application of attentional focus strategies, External Focus of Attention (EFA) aiming at the movement effect has been reported to have more efficacy than Internal Focus of Attention (IFA) aiming at movement characteristics in healthy subjects. There are not many studies that compare the EFA and IFA instructions in people with Musculoskeletal (MSK) and Central Nervous System disorders (CNS). The purpose of this systematic review is to determine if IFA or EFA, in patients with CNS or MSK, may improve performance and have some effects on motor learning. Databases used for research: PubMed, CINAHL, Cochrane Library, PEDro, PsycINFO, SCOPUS. Inclusion criteria: Randomized Controlled Trial, quasi-Randomized Controlled Trial, enrolled subjects with CNS or with MSK and compared the efficacy of EFA and IFA. The studies suggest that the EFA is better than IFA in affecting the movement execution in patients with MSK, while conflicted findings emerge in presence of CNS disorders. Studies included in the qualitative analysis showed heterogeneous methodological features in study design and conductance, so results must be interpreted with caution.

## 1. Introduction

Attentional focus is an important factor influencing motor task execution, associated with accuracy and reliability in achieving the goal (efficacy), as well as fluent and economical movement executions and automaticity, as evidenced by the investment of relatively little physical and mental effort (efficiency) [1,2,3]. Furthermore, it also influences all the phases of motor performance and learning (acquisition, retention, transfer) [4]. The acquisition phase refers to the early task presentation where the attention will be directed to each element or sequence of the movement, developing a mental representation of the task. In the retention phase, the mental representation is gradually improved with practice up to a semi-stabilization phase over time. The transfer phase refers to the impact that established skills have in favoring the acquisition of new skills or in promoting the task performance in a different context [4].

To influence motor performance and learning, researchers and clinicians usually use several cognitive facilitations such as verbal instructions given before or during the execution of the motor task [1]. The verbal instructions have to direct the focus of attention on a specific attentional source: an Internal Focus of Attention (IFA) or an external focus of attention (EFA) [5]. The IFA is referred to the attention focused on an anatomic segment, a joint, a corporeal reference or a sensorial stimulus. The EFA involves the focusing of the attention toward the movement effects or on a specific instrument used for task [5]. For example, during the darts throwing, the patient should focus on the middle of the target (EFA) or on the movements of fingers, wrist and elbow (IFA) [6].

Several researches showed that EFA is more beneficial to movements efficiency and efficacy than IFA in healthy subjects [1]. Indeed, EFA improved motor performance and learning in different laboratory tasks and sport skills such as standing balance, golf, volleyball, soccer kick, dart throwing, baseball, tennis, jump, basketball, running, force production, postural and supra-postural task and oral-motor task [1,7,8].

The *Constrained Action Hypothesis*, proposed by Wulf, McNevin and Shea (2001), may explain the effects of attentional focus strategies [9]. In accordance with this hypothesis, IFA would require an increased conscious control of the movement that could interfere with the usual automatic motor control processes. At opposite, EFA reduces the need of conscious control of the movement, favoring the automatic processes of motor control, thus resulting in better performance and learning [9].

The advantages of EFA, compared to IFA, can be described at different levels of neural motor system. In a functional magnetic resonance (fMRI) study, EFA increased the activation of the primary motor cortex and the primary somatosensory cortex insular region, during a task that required the execution of a sequence with the fingers [10]. Furthermore, using a single-pulse transcranial magnetic stimulation at intensity below the motor threshold and paired-pulse transcranial magnetic stimulation, EFA activates GABAergic cortical inhibitory neurons, inducing a short intra-cortical inhibition interval (SICI) [11,12,13]. Moreover, EFA induces a reduction of electromyographic activity during motor tasks, thus enhancing the overall movement economy [14,15,16,17].

In the last twenty years, the emerging evidence observed in healthy population has led the rehabilitation community to debate about the clinical role of attentional focus [18]. Up to now, only two systematic reviews have investigated the effect of focus of attention on motor performance and learning in motor impairments [19,20]. In 2011 Rossettini et al., revealed that EFA improves performance and motor learning better than IFA in patients with Central Nervous System (CNS) and Musculoskeletal (MSK) disorders [19]. In 2013, Sturmberg et al. [20], considering only patients with MSK disorders, reported that EFA increases motor performance, without affecting pain and function.

Since the publication of these reviews new narrative reviews and primary studies were published [20,21,22] and there is currently a need for an update. Indeed, new published studies with significant results may potentially change conclusions reached previously, thus influencing clinical practice in rehabilitation [23,24]. 

The aim of this systematic review is to collect more recent scientific evidence to update the preceding reviews.

## 2. Materials and Methods 

### 2.1. Protocol and Eligibility Criteria

To set up a correct methodology, the review was written according to the PRISMA (Preferred Reporting Items for Systematic Reviews and Meta-Analyses) guidelines [25,26]. The authors of this systematic review have an extensive experience in performing systematic reviews; own specific clinical expertise in rehabilitation of motor impairments in patients with CNS and MSK disorders.

Eligibility criteria were as follows: Randomized Controlled Trial (RCT) or quasi-Randomized Controlled Trial (q-RTC); studies enrolling female and male subjects with any type of diagnosis of MSK or CNS disorders and between 18 and 90 years of age, studies comparing the efficacy of EFA based on verbal instructions (target-related) and the IFA based on verbal instructions (movement-related) during therapeutic exercise. Moreover, studies had to report the follow-up data of the outcomes during the retention phase (e.g., immediate, days, weeks) or the transfer phase (e.g., new context). Outcomes regarding both movement dynamics (e.g., kinematic and kinetic variables) and movement effects (e.g., task performance variables) were considered.

Exclusion criteria were as follows: observational studies, cross-sectional studies, studies considering attentional focus for feedback strategy purpose, studies analyzing physiotherapist’s or patient’s preference when applying attentional focus strategies, studies without a clear description of attentional source or patient’s diagnosis.

### 2.2. Data Sources, Search, and Study Selection

The research started in June 2017 and finished in August 2017. The research databases were: PubMed, CINAHL, Cochrane Library, PEDro, PsycINFO, SCOPUS. This study is different from the previous research [19], as SCOPUS has been added and EMBASE has been removed.

Keywords and search strategy for PubMed, Cochrane Library, SCOPUS, PsycINFO, CINHAL were as follows:

(“attentional focus” OR “focus of attention” OR “attentional focusing” OR IFA OR EFA OR “External focus” OR “internal focus”) AND (performance OR balance OR “motor learning” OR instruction *).

The keywords and search strategy have been changed compared to the previous study [19] that only reported: “attentional focus” OR “focus of attention” OR “attentional focusing”. No other filters/limits for language and study design were used during the research phase.

All duplicates were eliminated after exporting all articles from databases using reference manager Mendeley Desktop (Mendeley Ltd.; v.1.18, www.mendeley.com) and afterwards hand checked to remove the errors possibly due to the software. During the screening phase, two researchers (Alessandro Piccoli, Giacomo Rossettini) independently screened titles and abstracts of identified records for inclusions or exclusion eligibility process. If an abstract was relevant, then the full text was retrieved and assessed for eligibility according to exclusion and inclusion criteria. If these two reviewers did not agree to a specific study, a third-party author (Simone Cecchetto) made the final decision. 

Computation of agreement between reviewers regarding inclusion and exclusion of studies in eligibility process was carried out by assessing the percentage of agreement [27]. Microsoft^®^ Excel^®^ (v.1804, Microsoft Corporation, Redmond, WA, USA) was used for computation of agreement.

### 2.3. Data Collection Process

Two reviewers (Alessandro Piccoli, Giacomo Rossettini), separately, registered manually the data in specific charts and finally the data was cross-checked. The data involve authors, study design (RCT, q-RCT, Crossover Trial), subjects (numbers, age, gender, diagnosis) therapeutic exercise protocol (task type, trial numbers, days, execution condition, source of attention) outcomes (kinematic, kinetic, performance variations) and follow-up (retention and transfer phase). The main outcome results concerning the comparison between EFA and IFA were extracted from the studies performed by two authors (Antonello Viceconti, Diego Ristori). If there were not any conclusions after the text comparison, they obtained the results using tables or figures.

### 2.4. Critical Appraisal of Studies

The methodological quality was assessed with the PEDro scale developed by the “Centre for Evidence-Based Practice” in Australia based on general core criteria used for RCT and q-RCT [28,29]. The validity [30] and reliability [31,32] of the PEDro scale were amply recognized. PEDro scale is a dichotomous 11-items scale having a total score of 10 points. Every item assesses a study methodological feature that may be assessed as present or absent. Item regards eligibility criteria (1 item, not considered in the overall score), randomization methods (2 items), blinding methods (3 items), data report (3 items) and data analysis (2 items). 

All the eligible articles were included in statistical analysis, irrespective of the critical appraisal results. Studies were classified according to the following criteria: “excellent” (PEDro 9–10), “good” (PEDro 6–8), “fair” (PEDro 4–5), “poor” (PEDro 0–3) [33]. Two assessors (Andrea Turolla, Filippo Maselli), after a training period of six months, performed separately the methodological critical appraisal. A third assessor (Marco Testa) solved disagreement between reviewers and made the final decision. 

Computation of agreement between reviewers regarding critical appraisal of single studies was analyzed by the percentage of agreement [27]. To calculate the agreement for inclusion of studies three categories were identified: “not considered”, “removed”, and “accepted”. Microsoft^®^ Excel^®^ (v.1804, Microsoft Corporation) was used for computation of agreement.

## 3. Results

### 3.1. Study Selection

**Figure 1:**
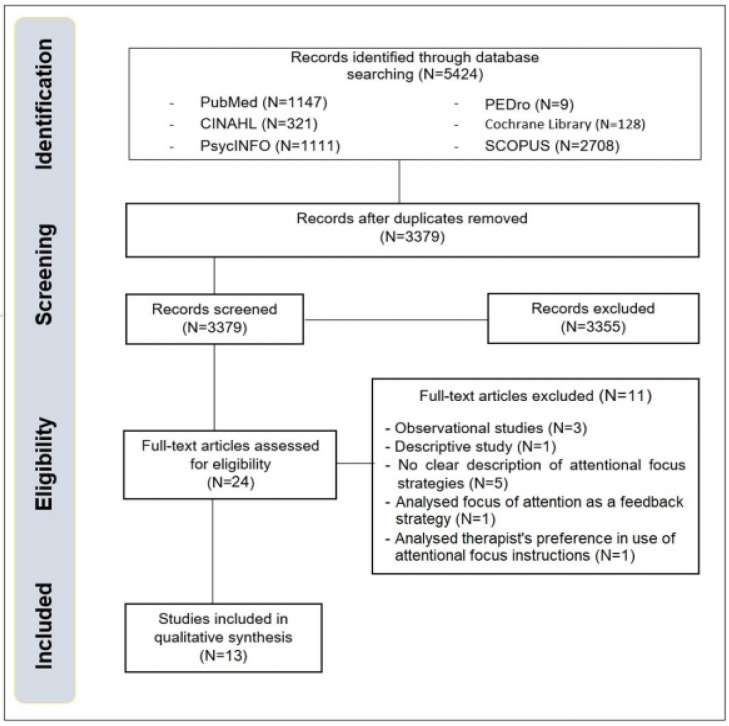
PRISMA flow diagram.

The research process produced 5424 results, resulted in 3379 records after the elimination of duplicated data. After the screening process, for titles and abstracts, 24 full-text studies were retrieved. During the eligibility process 11 studies were eliminated (Table A1, Appendix A) because of: observational studies [34,35,36], descriptive studies [37], no clear strategy to direct the attentional focus [38,39,40,41,42], attentional focus as feedback strategy [43], analysis of therapist’s preference when applying attentional focus [44]. 13 studies finally satisfied the inclusion criteria. Considering patients with CNS disorders [45,46,47,48,49,50,51,52,53,54] and patients with MSK disorders [55,56,57]. The PRISMA flow diagram representing the screening process is reported in Figure 1.

The inter-reviewer agreement of the eligibility process was the 99.9%.

### 3.2. Study Characteristics

Considering the participants’ sample, one RCT and four q-RCT enrolled 150 people with sub-acute stroke [45,49,50,51,54], one q-RCT enrolled 28 people with acute stroke [53], one RCT and three q-RCT enrolled 79 people with Idiopathic Parkinson [46,47,48,52], a RCT enrolled 16 people after anterior cruciate ligament reconstruction (ACL) [57] and two RCT enrolled 76 people with ankle sprain [55,56]. The sample size was limited in all the studies (16–42 subjects) and there were heterogeneous ages. The 349 subjects enrolled in this study included 81 patients allocated to the EFA group and 88 to the IFA group. The remaining 180 received both focus type with a different order.

The studies compared verbal instructions that direct the focus externally with verbal instructions that direct the focus internally in these main tasks: balance task [46,47,52,55,56]; lateral body weight shift [50]; darts throw [48]; reach-to-grasp [45,49]; single leg stepping [51]; tracing a trajectory [53,54]; single leg jump [57]. On average, the studies had duration of 1.54 days (range: 1–2 days) with 27 repetitions (range: 3–96 repetitions). Only one study presented a longer duration (28 days) and a higher number of repetitions (max 960) [54].

Outcomes considering the task performance variables were the immediate body weight shift, the anterior-posterior deviation of the center of mass and the maximum anterior, posterior and horizontal center of mass [47,50,52,55,56], equilibrium score [46], mean radial error [48], movement time, movement units, peak velocity, time to peak velocity, percentage of time to peak velocity, movement fluency, dual-task costs [45,51,53]. Moreover, studies reported outcomes about time to peak deceleration, percentage of time to peak deceleration, peak aperture size, time to peak aperture and percentage of time to peak aperture [49], hand movement velocity, hand movement error [53], mean jump distance, time to peak valgus angle, time to peak flexion angle [57].

Outcomes considering the kinematic and kinetic variables were peak elbow extension, peak shoulder flexion and peak trunk flexion [49], knee flexion angle at initial contact, peak knee flexion angle, flexion range of motion (ROM), knee valgus angle at initial contact, peak knee valgus angle, valgus ROM [57], joint independence [54].

In one of the studies patients were followed up during the retention phase (5 min later the repetition) [49], two studies did the follow-up during the retention phase 24–48 h after the acquisition phase [48,55], another one during the transfer phase 24–48 h after the acquisition phase [48,56] and the last one during retention phase, 4 weeks after discharge [54]. Eight studies did not carry out follow-up measures but only analyzed the acquisition phase. 

Studies characteristics are listed in Table A2, Table A3 and Table A4. (Appendix A).

### 3.3. Critical Appraisal of the Studies

All the studies have a good external validity and a good statistical method. Three studies present a good internal validity [48,50,55], four studies have a fair internal validity [49,54,56,57] while the other research findings present a poor internal validity [45,46,47,51,52,53]. The item regarding the physiotherapists’ blindness was absent in all the studies. Based on the score, five studies were classified as good [48,49,50,54,55], three as fair [53,56,57] and five as poor [45,46,47,51,52]. Regarding the methodological assessment, the inter-reviewer percentage agreement was 98%.

The methodological quality of studies is presented in Table 1.

### 3.4. Effect of Attentional Focus on CNS Disorders

#### 3.4.1. Stroke

In patients with stroke, Fasoli et al. [45], used the attentional focus effects during three reaching tasks: removing a can from a shelf (task A), putting an apple into a basket (task B) and moving a coffee mug onto a saucer (task C). EFA, in comparison to IFA, reported a significant shorter movement time (task A *p* = 0.002, task B *p* = 0.054, task C *p* = 0.027) and greater peak velocity (task A *p* = 0.002, task B *p* = 0.035, task C *p* = 0.013). However, movement unit, which is the combination of acceleration and deceleration phases of reaching task, suggested a greater efficacy (large effect size) of EFA instructions only in one task (*p* = 0.019).

With the same kind of patients (stroke), Durham et al. [49], investigated the effects of the different attentional focus in three different tasks: reaching to grasp a jar (task A), placing a jar forwards on to a table (task B) and placing a jar on a 28 cm wooden platform (task C). During the task A, there was a significant increase of percentage time to peak velocity (*p* = 0.039) for EFA in comparison to IFA. During task B, EFA reported a less movement duration (*p* = 0.008) and an increase in percentage time of deceleration (*p* = 0.01). Moreover, there was a significant three-way interaction when IFA came first than EFA. In task B, there was a reduction in the mean movement duration (*p* = 0.018) and a significant increase in the mean percentage of time to peak aperture (*p* = 0.04). In task C there was an increase of the mean time to peak deceleration (*p* = 0.017).

During the lateral body weight shift toward the healthy side in people with stroke, in the study of Mückel and Mehrholz [50], the EFA improved their maximum distance in lateral body weight shift significantly more than IFA (8.7 cm ± 2.6 vs. 4.5 cm ± 3.3; *p* = 0.006); significant differences were not found between the groups in anterior-posterior deviation during the task (2.3 cm ± 1.3 vs. 1.2 cm ± 1.2; *p* = 0.085). In a post hoc regression analysis, it was not found any association of baseline variables with the effect of immediate lateral body weight shift.

Kal et al. [51], analyzed the effect of different focus instructions during single-task (single leg stepping) and dual-task (single leg stepping + auditory reaction time or letter fluency). During single-task there was not significant difference in movement speed between the two attentional strategies (*p* = 0.341) and there was not significant interaction between focus and leg (*p* = 0.387). The authors also analyzed if there was a possible interaction between focus and different factors such as cognitive capacity (level of education and Mini Mental State Examination), Motricity Index score, Fugl-Meyer score and Movement-Specific Reinvestment Scale score. 

Findings revealed that patients with higher Fugl-Meyer score improved movement speed using EFA compared to IFA (β = 2.32). Moreover, patients with higher movement reinvestment score presented a decrease in movement speed in EFA condition than in IFA condition. Concerning movement fluency, there was no difference in attentional strategies (*p* = 0.644). Measured the dual-task costs (DTCs), IFA showed generally leaning to lower DTCs compared with EFA but was not significant (*p* = 0.065). Subsequent effect modification analyses revealed there was an interaction between focus and attention domain score, better attentional capacity seemed to reduce DTCs in EFA (β = −2.98) than IFA (β = −0.62). Finally, movement fluency did not differ between EFA condition and IFA condition (*p* = 0.132).

Sakurada et al. [53], analyzed the attentional focus efficiency in patients with acute stroke. First, assessed motor imagery abilities to identify the dominance, visual or kinesthetic. Subjects then performed a visuomotor task that required tracing a trajectory under three attentional conditions (no instruction, IFA, EFA). Subjects with visual dominance made significantly more movement errors than those with kinesthetic dominance under the IFA condition (*p* = 0.044). Moreover, subjects with visual dominance showed more accurate movement under the EFA condition, while patients with kinesthetic dominance showed more accurate movement under the IFA condition. Regarding hand velocity, EFA is better in both groups (*p* = 0.0009).

Kim et al. [54] analyzed the effect of different focus of attention on joint independence (JI), Fugl-Meyer Assessment upper extremity subscale (FMA), Wolf Motor Function Test (WMFT) and Manipulation Check Questionnaire (MCQ) in a task that required to move a virtual ball toward a target in an 8-point clock pattern, with InMotion ARM™ and demonstrated significant differences in within-group for JI-EFA (*p* < 0.0005) and JI-IFA (*p* < 0.0005). Moreover, the post hoc test reported a significant improvement for JI-EFA and JI-IFA from baseline to discharge (*p* = 0.002; *p* < 0.0005) and baseline to follow-up (*p* = 0.001; *p* < 0.0005), but not from discharge to follow-up (*p* = 0.461). Scores of WMFT and FMA were significant within-groups across time (*p* < 0.0005), with a post hoc test indicated significant improvement from baseline to discharge (WMFT: *p* = 0.002; FMA: *p* < 0.0005) and from baseline to follow-up (WMFT: *p* < 0.0005; FMA: *p* < 0.0005). Finally, the MCQ did not report any statistically significant difference between groups.

Efficacy of attentional focus in motor learning of subjects with stroke is presented in Table 2.

#### 3.4.2. Idiopathic Parkinson

Landers et al. [46], analyzed three postural control tasks in subjects with Idiopathic Parkinson and they showed the absence of a significant difference between the three focus conditions (no instructions, EFA and IFA) during the retention phase. However, after the post hoc test, a subgroup analysis revealed a significantly improved performance in fallers subgroup subjects (participants with a reported history of falls) treated with EFA (*p* < 0.05). This improvement is present in many conditions.

With a similar sample, Wulf et al. [47], analyzed the attentional focus effects during postural tasks using an unstable surface (balance disk). Participants were instructed to reduce the movements of their feet (EFA) or of the disk (IFA). EFA reported a significant reduction of postural sway during the retention test in comparison to IFA (*p* < 0.05).

Kakar et al. [48], analyzed darts throw task in subjects with Idiopathic Parkinson and compared the effects of the different focus during the acquisition, retention and transfer phase dividing them in different blocks (acquisition = 5 blocks × 10 throws, retention = 2 blocks × 10 throws, transfer = 2 blocks × 10 throws). During the acquisition phase, there are not any differences between the first four blocks (*p* > 0.05). Instead, in the fifth block, there is a reduction in EFA mean radial error (*p* = 0.004) regarding the IFA. During the retention phase, EFA has a mean radial error lower than IFA but it is not statistically significant (*p* = 0.052 [block 1], *p* = 0.11 [block 2]). Finally, during the transfer phase, EFA has a mean radial error lower in both blocks (*p* = 0.003 [block 1], *p* = 0.006 [block 2]) in comparison to IFA.

Beck and Almeida [52], analyzed the efficacy of the two attentional focus during postural tasks using an unstable surface (Biodex Balance System) in subjects with Idiopathic Parkinson during ON (presence) or OFF (absence) dopamine replacement medication. During OFF medication, IFA had a less anterior-posterior sway in comparison to the control group (no instructions, *p* = 0.02) or to the EFA (*p* = 0.04); there was no difference in medial-lateral sway. Moreover, sway displacement and variability were significantly lower in the IFA condition when OFF medications compared with the IFA (*p* < 0.01) and EFA (*p* < 0.01) conditions while on medications.

Efficacy of attentional focus in motor learning of subjects with Idiopathic Parkinson is presented in Table 3.

### 3.5. Effect of Attentional Focus on MSK Disorders

#### 3.5.1. Ankle Sprain

Rotem-Lehrer & Laufer [56] analyzed subjects with ankle sprain during a balance task. The EFA group showed a better performance during the transfer phase at 48 h follow-up (*p* < 0.05). There was no significant difference in the pre-post-training score over time for IFA group members in all outcomes (overall stability index (OSI), anterior-posterior stability index (APSI), medium-lateral stability index (MLSI)). The group by time interaction was statistically significant for all outcomes (OSI *p* = 0.001, APSI *p* = 0.03, MLSI *p* = 0.01).

Laufer et al. [55], found that there were similar long-term results in the similar sample during the retention phase. In this study, the postural control was tested under two different conditions: more stable conditions (level 6 on 8) and less stable position (level 4 on 8). Only the group under the EFA realized a statistically significant improvement during the pre-training and post-training in the most stable position. The improvement happened for two of the three outcomes (OSI *p* = 0.030, APSI *p* < 0.001). The group by time interaction was statistically significant in OSI (*p* = 0.030) and APSI (*p* = 0.019). In the least stable position, both groups experienced a significant improvement in OSI and APSI but no significant interaction effects between group and time was revealed. About the MLSI there was no any difference in both stability conditions.

Efficacy of attentional focus in motor learning of subjects with ankle sprain is presented in Table 4.

#### 3.5.2. Anterior Cruciate Ligament Reconstruction

Gokeler et al. [57] analyzed the attentional focus effect during the single leg jump in subjects after anterior cruciate ligament (ACL) reconstruction. The EFA was better than IFA for different outcomes. As for the injured leg, there was a significantly smaller knee flexion at initial contact in the IFA group compared to the EFA group (EFA 37.38° ± 6.44° vs. IFA 27.25° ± 11.09°; *p* = 0.04). Peak knee flexion was significantly lower in the IFA group for the non-injured legs compared to the EFA group (IFA 51.63° ± 12.93° vs. EFA 69.26° ± 12.21°; *p* = 0.01) and for the injured legs (IFA 51.75° ± 16.67° vs. EFA 69.54° ± 11.44°; *p* = 0.01). The IFA group’s time to peak knee flexion, for the non-injured leg, compared to the EFA group was significantly shorter (EFA 0.21 s ± 0.04 s vs. IFA 0.16 s ± 0.03 s, *p* = 0.01). Time to peak knee flexion for the injured legs was significantly shorter for the IFA group compared to the EFA group (EFA 0.21 s ± 0.03 s vs. IFA 0.16 s ± 0.05 s, *p* = 0.02). The analysis of the other outcomes did not show any statistically significant difference.

Efficacy of attentional focus in motor learning of subjects with ACL reconstruction is presented in Table 4.

## 4. Discussion

### 4.1. Effect of Attentional Focus on Performance and Motor Learning

This systematic review updates the results of previous reviews performed by Rossettini et al. 2011 [19] and Sturmberg et al. 2013 [20] and shows conflicting evidence about the use of the optimal attentional strategy in patients with CNS disorders, compared to healthy subjects [1,19,20]. Indeed, in presence of motor impairments such as stroke [45,49,50,51,53,54] and Idiopathic Parkinson [46,47,48,52], EFA did not always improve motor performance and learning compared to IFA giving to the rehabilitative community the opportunity to reflect about different factors capable to impact on motor task execution.

In accordance with the Constrained Action Hypothesis [9], EFA improves performance in motor tasks such as lateral body weight shift [50] and lower limb movement [51] compared to IFA, thus facilitating the automatic motor control processes while studies analyzing the reaching and the manipulation of an object report contradictory effects of attentional focus instructions [45,49,53,54]. The sensory process involved in motor task [58], the scheduling of practice [59], the patient’s preference toward a specific type of motor imagery [53] and the severity of motor impairments [54] could explain the heterogeneity of results in upper limb performance.

Indeed, during the planning and the execution of motor task, the attentional strategies activate different sensory processes [60]. While EFA emphasizes the visual channel, collecting salient information regarding the object/target; IFA favors afferent information of movement through the proprioceptive channel [60]. In patients with stroke, the visualization, because of the proprioceptive impairment, represents a predominant strategy and helps to compensate for the proprioceptive deficit during the upper limb motor task [58]. Moreover, the benefit of EFA is amplified if it is preceded by tasks adopting IFA instruction during rehabilitation [49]. Indeed, patients with stroke present difficulties during processing of implicit information such as execution of a motor task without any information on how a limb should move [59,61]. Therefore, provide explicit information (IFA) first could help patients to analyze the subsequent implicit information adopted when EFA instructions are delivered [60]. Also, the patient’s preference toward a specific type of motor imagery (e.g., visual and kinesthetic) influences differently the effects of the attentional focus [53]. Visual motor imagery, involving mental processes for the visualization of patients’ body movements, seems to benefit more from EFA instructions compared to IFA instructions while kinesthetic motor imagery, simulating the feeling of muscle or joint sensations, seems to benefit more from IFA instructions [53]. The severity of motor impairments influence the effectiveness of attentional focus instructions especially on upper limb motor performance [54]: the benefit of EFA, compared to IFA, is missing in patients with a more severe arm impairment, probably due to the severity of sensory impairment and of paresis, that limited the automatic processes which regulate motor control [54].

According to the Constrained Action Hypothesis [9], this systematic review confirms the benefit of EFA, compared to IFA, in patients with Idiopathic Parkinson during upper limb motor task [48] as an attentional source capable to promote automatic and self-regulated processes. Differently from what found for healthy subjects [1], our results displayed conflicting findings about the effect of EFA in task balance [46,47,52], explainable by different factors such as history of fall [46] and presence/absence of dopaminergic medication [52].

Patients with Parkinson usually report balance problems, anxiety, fear disturbances and unexpected falls [62,63]. The history of falls, associated to the worsening of patients’ degree of instability, makes the balance task more complex and favor EFA instructions [46]. Indeed, the effects of EFA are enhanced in case of more complex tasks requiring high effort [64] as fallers compared to non-fallers. 

The presence/absence of dopaminergic medication may explain the different effects of attentional strategies associated to balance [65,66]. Several studies [46,47] showed that EFA instruction, compared to IFA, improves postural stability, thus decreasing anxiety and fear of falling [67] only when patients are taking dopamine medication (ON phase) [52]. This observation implies that, when taking dopamine, patients with Idiopathic Parkinson are able to recruit and utilize automatic processes by EFA, despite the pathological degeneration of automatic pathways [52]. When dopamine is depleted (OFF phase), the adoption of EFA strategies, compared to IFA, induces a worsening of postural stability and balance [52]. Instead, IFA improves balance probably because of the higher activation of frontal cortical areas for control of movement and lower involvement of the impaired basal ganglia [52].

Considering patients with MSK disorders such as ankle sprain [55,56] and ACL reconstruction [57], this systematic reviews reported that EFA improves motor performance and learning better than IFA.

In accordance with the Constrained Action Hypothesis [9], this finding confirms the results observed in healthy subject during balance and jump task [1]. Indeed, in patients with MSK disorders, EFA promotes the use of unconscious or automatic processes, thus facilitating the execution of motor task. Instead, IFA results in a more conscious type of control that constrains the motor system and disrupts automatic control processes [9]. Furthermore, EFA compared with IFA, could represent a safety strategy to prevent re-injuries, quite common after ankle sprain [68] and ACL reconstruction [69,70,71], thus resulting useful during rehabilitation of the landing phase of a jump [72]. 

Finally, the success of attentional focus instructions could be influenced also by the physiotherapist’s preference and habit in adopting specific source of attention. Indeed, in daily practice, physiotherapists adopt instructions and feedbacks that direct the attention more frequently to the patient’s body (IFA) [35,44] or to a mixed focus of attention (IFA, EFA or non-specific) [36], thus influencing a possible preference for a specific attentional source. To avoid this possible source of bias, physiotherapists should investigate the patient’s preference and skills’ level in a specific task before deciding which instruction could represent the optimal strategy for motor performance and learning for the individual patient [73].

In this systematic review, the impact of attentional focus strategies in both patients with CNS [45,46,47,48,49,50,51,52,53,54] and MSK [55,56,57] disorders is not moderated by age and gender factors. This finding confirms that the effect is related to the adopted source of attention (EFA, IFA) rather than to other factors both in healthy subjects and in patients with motor impairments [1,7,8,74].

### 4.2. Limits of Systematic Review

A common problem with every systematic review process is that publication bias could challenge result validity. Studies that do not demonstrate any benefits are less likely to be published, therefore creating a publication bias. Even if this revision was elaborated using 6 different research databases, from inception to August 2017, including different languages, some relevant articles may be excluded determining a possible difference of results. Furthermore, gray literature has not been included (gray literature bias), thus risking to exclude possible studies [75,76]. Only four studies reported short time retention and transfer test. However, longer follow-up is needed to increase the knowledge about stabilization process of learned imprint during attentional focus [60]. Moreover, most of the selected studies used no clinically relevant outcomes measures; this allows updating the evidence but not their transposition into clinical practice. Finally, a reporting bias could have occurred because the review was not registered on PROSPERO (an international prospective register of systematic reviews).

## 5. Conclusions

### 5.1. Implications for Research

This revision was developed with the aim to update and collect more recent evidence regarding attentional focus strategies efficacy using verbal instructions in motor learning and performance in subjects with MSK and CNS disorders.

There is a need of studies with sound methodological quality, enrolling subjects with different disorders affecting both the CNS and MSK system, adopting complex tasks and outcomes measures with a more clinical relevance and longer follow-up periods. These represent the necessary conditions to evaluate the impact that attentional focus strategies have on the cost/benefit ratio of re-learning training.

### 5.2. Implications for Clinical Practice

This systematic review suggests that the EFA is better than IFA in affecting motor performance and learning in patients with MSK disorders, while conflicting findings emerge in presence of CNS disorders. These conclusions may lead the decisional process to the use of attentional focus in clinical practice. However, caution is needed when interpreting the findings of this review due to the large heterogeneity and the methodological quality of the retrieved studies. Data from this review offer additional information about the potential use of attentional focus during therapeutic exercise planning; nevertheless, the final decision should also consider the physiotherapist’s expertise and patient’s preference.

## Figures and Tables

**Table 1:** Critical appraisal of studies.

Study	Eligibility Criteria	Subject Random Allocation	Concealed Allocation	Comparability at Baseline	Blinding Subject	Blinding Therapist	Blinding Assessor	Follow-up Evaluations	Intention-to-Treat Analysis	A Between-Group Statistical Comparison	A Point Measure	Score	Quality
Fasoli et al. [45]	1	1	0	0	0	0	0	0	0	1	1	3/10	Poor
Landers et al. [46]	1	0	0	0	0	0	0	0	0	1	1	2/10	Poor
Wulf et al. [47]	1	1	0	0	0	0	0	0	0	1	1	3/10	Poor
Kakar et al. [48]	1	1	0	1	0	0	1	1	1	1	1	7/10	Good
Durham et al. [49]	1	1	0	1	0	0	0	1	1	1	1	6/10	Good
Mückel & Mehrholz [50]	1	1	1	1	1	0	1	0	0	1	1	7/10	Good
Kal et al. [51]	1	0	0	0	0	0	0	0	0	1	1	2/10	Poor
Beck & Almeida [52]	1	1	0	0	0	0	0	0	0	1	1	3/10	Poor
Sakurada et al. [53]	1	1	1	0	0	0	0	0	0	1	1	4/10	Fair
Kim et al. [54]	1	1	0	1	0	0	1	1	0	1	1	6/10	Good
Laufer et al. [55]	1	1	0	1	0	0	1	1	1	1	1	7/10	Good
Rotem-Leherer & Laufer [56]	1	1	0	1	0	0	1	0	0	1	1	5/10	Fair
Gokeler et al. [57]	1	1	1	1	0	0	0	0	0	1	1	5/10	Fair

**Table 2:** Effects of attentional focus instructions on Stroke.

Study	Outcome	Post Hoc Test (EFA vs. IFA) or Anova
Fasoli et al. [45]	MT PV MU %TPV	Significant improvement during all three tasks executed when given EFA instructions vs. IFA instructions (*p* < 0.05). Significant improvement during one task when giving EFA instructions vs. IFA instructions (*p* = 0.019). No difference between EFA and IFA groups (*p* > 0.05).
Durham et al. [49]	MD PV TPV %TPV TPD %TPD PAS TPA %TPA PEE PSF PTF	During task A, a significantly increased %TPV (*p* = 0.039) was found using EFA compared with IFA. EFA group had significantly less MD (*p* = 0.008) and increased %TPD (*p* = 0.01) during task B. Significant interaction effects between focus and order were found (IFA → EFA). Significant reduction in MD (*p* = 0.0018) and increase in %TPA (*p* = 0.04) during task B. Significant increase in TPD (*p* = 0.0017) during task C. No significant difference for the other outcomes.
Mückel & Mehrholz [50]	IBWS APCOM	Significant improvement in IBWS with EFA, compared with IFA (*p* = 0.006). No significant difference in APCOM (*p* = 0.085).
Kal et al. [51]	MS MF DTCs	During single-task there was not significant difference in movement speed between two attentional strategies (*p* = 0.341). There was not significant interaction between focus and leg (*p* = 0.387). Higher Fugl-Meyer score showed improvements in movement speed in EFA condition than IFA condition (β = 2.32). Higher movement reinvestment score showed decreases in movement speed in EFA condition than in IFA condition. IFA showed generally leaning to lower DTCs compared with EFA but was not significant. Better attentional capacity seemed to reduce DTCs in EFA (β = −2.98) than IFA (β = −0.62).
Sakurada et al. [53]	HME HV	Subjects with visual dominance showed more accurate movement under the EFA condition, while patients with kinesthetic dominance showed more accurate movement under the IFA condition. EFA showed a significantly faster hand velocity in both groups (visual and kinesthetic dominance; *p* = 0.0009).
Kim et al. [54]	JI FMA WMFT MCQ	There were no between-group differences for JI at discharge and follow-up. There were significant differences in within-group for JI-EFA and JI-IFA from baseline to discharge and baseline to follow-up (*p* < 0.0005; *p* < 0.0005). WMFT and FMA there were not between-group significant statistically difference at discharge to follow-up. Scores of WMFT and FMA were significant within-groups across time (*p* < 0.0005). The MCQ did not report any statistically significant difference between-group.

MT: movement time; PV: peak velocity; MU: movement units; TPV: time to peak velocity; %TPV: percentage of time to peak velocity; MD: movement duration; TPD: time to peak deceleration; %TPD: percentage of time to peak velocity; PAS: peak aperture size; TPA: time to peak aperture; %TPA: percentage of time to peak aperture; PEE: peak elbow extension; PSF: peak shoulder flexion; PTF: peak trunk flexion; APCOM: anterior-posterior center of mass; IBWS: immediate body weight shift; MS: movement speed; MF: movement fluency; DTCs: dual-task costs; HME: hand movement error; HV: hand velocity; JI: joint independence, FMA: Fugl-Meyer Assessment, WMFT: Wolf Motor Function Test; MCQ: Manipulation Check Questionnaire.

**Table 3:** Effects of attentional focus instructions on Idiopathic Parkinson.

Study	Outcome	Post Hoc Test (EFA vs. IFA) or Anova
Landers et al. [46]	ES	No significant EFA advantages for overall group (*p* > 0.05); benefits of EFA in fallers group under sway referenced condition (*p* < 0.05).
Wulf et al. [47]	COP	More-effective performance with the EFA than with IFA (*p* < 0.001).
Kakar et al. [48]	MRE	EFA had significantly less MRE for the acquisition phase (*p* = 0.004) and the transfer phase (*p* = 0.003 [block 1]; *p* = 0.006 [block 2]). EFA had a less MRE for the retention phase but the difference was not statistically significant (*p* = 0.052).
Beck & Almeida [52]	PSI	IFA group had a significantly lower anterior-posterior sway during OFF medication, compared control group (no instructions, *p* = 0.02) or to the EFA (*p* = 0.04). No difference in medial-lateral sway. IFA group had significantly lower sway displacement and variability during off medications compared with the IFA (*p* < 0.01) and EFA (*p* < 0.01) conditions during on medications.

ES: equilibrium score; COP: center of pressure displacement; MRE: mean radial error; PSI: postural stability index.

**Table 4:** Effects of attentional focus instructions on MSK disorders.

Study	Outcome	Post Hoc Test (EFA vs. IFA) or Anova
Laufer et al. [55]	OSI APSI MLSI	In the EFA group at the most stable position (level 6) increased efficacy was observed in the APSI (*p* < 0.001) and in the OSI (*p* = 0.030) stability index. At level 4 improvements were noted either in EFA and IFA group.
Rotem-Lehrer & Laufer [56]	OSI APSI MLSI	Significant improvement in all stability measures only in the EFA group (*p* < 0.05) while the IFA Group demonstrated a significant difference between pre- and post-training in only one stability measure. No significant difference between the groups (EFA and IFA) either pre-training or post-training.
Gokeler et al. [57]	MD KVAIC PKVA TPVA VA KFAIC PKFA TPF FA	Significant improvement of KFIC in the injured leg in the EFA group (*p* = 0.04). Significant improvement of PKF in both leg in the EFA (*p* = 0.01). IFA group TPKF was significantly shorter than EFA, in both legs (non-injured *p* = 0.01; injured *p* = 0.02). No significant difference for the other outcomes.

OSI: overall stability index; APSI: anterior/posterior stability index; MLSI: medium/lateral stability index; MD: mean distance; KVAIC: knee valgus angle at initial contact; PKVA: peak knee valgus angle; TPVA: time to peak valgus angle; VA: valgus angle; KFAIC: knee flexion angle at initial contact; PKFA: peak knee flexion angle; TPKFA: time to peak knee flexion angle; FA: flexion angle.

## Appendix A

**Table A1:** Studies excluded with motivations.

Study	Motivation
Impact of focus of attention instructions on walking performance in individuals with and without history of stroke. DePaul et al. 2009 [34]	Observational Study
Internal and EFA during gait re-education: an observational study of physical therapist practice in stroke rehabilitation. Johnson et al. 2013 [35]	Observational Study
How physical therapists instruct patients with stroke: an observational study on attentional focus during gait rehabilitation after stroke. Kal et al. 2017 [36]	Observational Study
The effects of attentional focus instructions on simulated upper extremity amputees’ movement kinematics when learning a novel functional task. McAlister 2006 [37]	Descriptive Study
Effects of single-task versus dual-task training on balance performance in older adults: a double-blind, RCT. Silsupadol et al. 2009 [38]	No clear strategy to direct the attentional focus
Interacting effects of cognitive load and adult age on the regularity of whole-body motion during treadmill walking. Verrel et al. 2009 [39]	No clear strategy to direct the attentional focus
Effects of different focus of attention rehabilitative training on gait performance in Multiple Sclerosis patients. Shafizadeh et al. 2013 [40]	No clear strategy to direct the attentional focus
Differences in attentional focus associated with recovery from sports injury: does injury induce an internal focus? Gray 2015 [41]	No clear strategy to direct the attentional focus
Does attentional focus during balance training in people with Parkinson’s disease affect outcome? A randomized controlled clinical trial. Landers et al. 2016 [42]	No clear strategy to direct the attentional focus
Improvement of arm movement patterns and endpoint control depends on type of feedback during practice in stroke survivors. Cirstea & Levin 2007 [43]	Attentional focus as feedback strategy
Use of information feedback and attentional focus of feedback in treating the person with a hemiplegic arm. Durham et al. *2009* [44]	Analysis of therapist’s preference when applying attentional focus

**Table A2:** Characteristics of the studies regarding patients with Stroke.

Intervention							
Study	Patient	Task	Condition	Instruction		Outcome	Follow-Up
				EFA (e.g.,)	IFA (e.g.,)		
Fasoli et al. [45] q-RCT	*N*. = 16 (both focus) Avg. Age = 61.2 years M/F = 10/6 Condition = stroke	Type = reaching N. trial = 8 Day = 1	-Seated position; use of right/left hand. -Seated position; use of right/left hand. -Seated position; use of right/left hand	“Put this can from the shelf on to the table. Pay attention to the can”. “Put this apple off a shelf into a basket. Pay attention to the apple”	“Put this can from the shelf on to the table. Pay attention to your arms” “Put this apple off a shelf into a basket. Pay attention to your arms”	MT PV MU %TPV	RT: / TT: /
Durham et al. [49] q-RCT	*N*. = 42 (both focus) Avg. Age = 61 years M/F = 30/12 Condition = Stroke	Task = Reach-to-Grasp N. trial = 96 Day = 1	-The jar was placed at 90% of arm’s length. -Task A: thumb and index finger placed together over a mark placed 15 cm from the table edge in a midline position. -Task B/C: hand grasping the jar that was placed on the same 15 cm midline marker	“With this straw I have taped on, can you ensure you keep close to it as you approach the jar?” “To grip well, you need to curl around the jar more”	“try and bring your wrist back as well.” “grip with your thumb and all of your fingers”	MD PV TPV %TPV TPD %TPD PAS TPA %TPA PEE PSF PTF	RT: 5 min TT: /
Mückel & Mehrholz [50] RCT	*N*.= 20 (10 IFA, 10 EFA) Avg. Age = 72.2 years M/F = 11/9 Condition = Stroke	Task = Lateral body weight shift N. trial = 3 Day = 1	-Patients sat on a sensor mat placed on a therapy bench, back unsupported. -External focus group: a green point was placed on the bench 20 cm lateral from the trochanter major of the ipsilesional trunk side	“Shift your body weight as much as possible toward the green circle without using your arms”	“Shift your body weight as much as possible toward your “healthy side” without using your arms”	IBWS APCOM	RT: / TT: /
Kal et al. [51]	*N*.= 39 (both focus) Avg. Age = 62.6 years M/F = 17/22 Condition = Stroke	Task = Single leg stepping N. trial = 24 Day = 3	-Seated comfortable, use of paretic/non-paretic leg. -A line was taped to the floor, in EFA conditions	“Alternately placing the foot in front of and behind the line”	“Alternately flexing and extending the leg”	MS MF DTCs	RT: / TT: /
Sakurada et al. [53] q-RCT	*N*.= 28 (both focus) Avg. Age = 64.9 years M/F = 10/18 Condition = Acute Stroke	Task = tracing a trajectory N. Trial = 30 Day = 1	-Seated on a chair or wheelchair in front of a desk with a monitor and wireless mouse -Distance between the participant’s eyes and the monitor was approximately 70 cm	“Direct attention to the cursor on the monitor”	“Direct attention to your hand movements”	HME HV	RT: / TT: /
Kim et al. [54] q-RCT	*N*. = 33 (18 IFA, 15 EFA) Avg. Age = 58.1 years M/F = 14/16 Condition = Stroke	Task = tracing a trajectory N. Trial = 960 max repetitions per session (12 session) Day = 28	-Seated with seat belt with shoulder/hip straps -In front of monitor -EFA affected arm was occluded from view	“Focus their attention at a video monitor and to move a yellow ball to various targets on the clock design”	“Focus their attention to the movement of their affected arm during training”	JI FMA WMFT MCQ	RT: 4 week TT: /

MT: movement time; PV: peak velocity; MU: movement units; TPV: time to peak velocity; %TPV: percentage of time to peak velocity; MD: movement duration; TPD: time to peak deceleration; %TPD: percentage of time to peak velocity; PAS: peak aperture size; TPA: time to peak aperture; %TPA: percentage of time to peak aperture; PEE: peak elbow extension; PSF: peak shoulder flexion; PTF: peak trunk flexion; APCOM: anterior-posterior center of mass; IBWS: immediate body weight shift; MS: movement speed; MF: movement fluency; DTCs: dual-task costs; HME: hand movement error; HV: hand velocity; JI: joint independence; FMA: Fugl-Meyer Assessment; WMFT: Wolf Motor Function Test; MCQ: Manipulation Check Questionnaire; RT: retention test; TT: transfer test.

**Table A3:** Characteristics of the studies regarding patients with Idiopathic Parkinson.

Intervention							
Study	Patient	Task	Condition	Instruction		Outcome	Follow-Up
				EFA (e.g.,)	IFA (e.g.,)		
Landers et al. [46] q-RCT	N. = 22 (both focus) Avg. Age = 72.7 years M/F = 17/5 Condition = idiopathic Parkinson (stage II or III)	Task = balance N. trial = 3 Day = 1	-Standing position; fixed support surface surround; eyes open -Standing position; fixed support surface; visual surround eyes closed -Standing position; surface sway referenced support and fixed surround; eyes open	“Put an equal amount of pressure on the rectangles” “Put an equal amount of pressure on the rectangles”	“Put an equal amount of for on your feet” “Put an equal amount of pressure on your feet”	ES	RT: / TT: /
Wulf et al. [47] q-RCT	N. = 14 (both focus) Avg. Age = 71.1 years M/F = 10/4 Condition = Idiopathic Parkinson (stage II or III)	Task = balance N. trial = 4 Day = 1	-Standing position; unstable surface	“Minimize movements of the disk”	“Minimize movements of your feet”	COP	RT: / TT: /
Kakar et al. [48] RCT	N. = 24 (12 IFA, 12 EFA) Avg. Age = 53.1 years M/F = 17/7 Condition = Idiopathic Parkinson (stage II or III)	Task = Throw Darts N. trial = 90 Day = 2	-1-m diameter circular target -Target height was 1.70 m, 3 m from the participant	“Look at the center of the board carefully for a few seconds.” “While throwing the dart, concentrate on its flight directly toward the target”	“Before throwing, concentrate on your finger motions and the correct position.” “While throwing, straighten all fingers simultaneously so that at the end of the throw, your hand is directed forwards and your elbow is fully straightened”	MRE	RT: 24 h TT: 24 h
Beck & Almeida [52] q-RCT	N.= 19 (both focus) Avg. Age = 71.4 years M/F = 17/2 Condition = Idiopathic Parkinson (stage II or III)	Task = Balance N. Trial = 18 Day = 2	-Stand over Biodex Balance System SD -On or off medication state	“Focus on minimizing the movements of the platform”	“Focus on minimizing the movements of their feet”	PSI	RT: / TT: /

ES: equilibrium score; COP: center of pressure displacement; MRE: mean radial error; PSI: postural stability index; RT: retention test; TT: transfer test.

**Table A4:** Characteristics of the studies regarding patients with MSK disorders.

Intervention							
Study	Patient	Task	Condition	Instruction		Outcome	Follow-Up
				EFA (e.g.,)	IFA (e.g.,)		
Laufer et al. [55] RCT	N. = 40 (20 IFA, 20EFA) Avg. Age = 20.8 years M/F = 36/4 Condition = Ankle Sprain (grade 1–2)	Task = balance N. trial = 20 Day = 4	-Standing position on unstable platform	“Keep your balance by stabilizing the platform”	“Keep your balance by stabilizing your body”	OSI APSI MLSI	RT: 48 h TT: /
Rotem-Lehrer & Laufer [56] RCT	N. = 36 (20 IFA, 16 EFA) Avg. Age = 20.9 years M/F = 36/0 Condition = Ankle Sprain (grade 1–2)	Task = balance N. trial = 20 Day = 4	-Standing position on unstable platform	“Keep your balance by stabilizing the platform”	“Keep your balance by stabilizing your body”	OSI APSI MLSI	RT: / TT: 48 h
Gokeler et al. [57] RCT	N. = 16 (8 IFA, 8 EFA) Avg. Age = 23.2 years M/F = 9/7 Condition = ACLr	Task = single leg jump N. trial = 5 Day = 1	-Stand on one leg -Land on the same leg	“Jump as far as you can. While you are jumping, I want you to think about pushing yourself off as hard as possible from the floor”	“Jump as far as you can. While you are jumping, I want you to think about extending your knees as rapidly as possible”	MD KVAIC PKVA TPVA VA KFAIC PKFA TPKFA FA	RT:0 h TT: /

ACLr: anterior cruciate ligament reconstruction; OSI: overall stability index; APSI: anterior/posterior stability index; MLSI: medium/lateral stability index; MD: mean distance; KVAIC: knee valgus angle at initial contact; PKVA: peak knee valgus angle; TPVA: time to peak valgus angle; VA: valgus angle; KFAIC: knee flexion angle at initial contact; PKFA: peak knee flexion angle; TPKFA: time to peak knee flexion angle; FA: flexion angle; RT: retention test; TT: transfer test.

## References

[1] Wulf G. (2013). Attentional focus and motor learning: A review of 15 years. Int. Rev. Sport Exerc. Psychol..

[2] Wulf G., Lewthwaite R. (2016). Optimizing performance through intrinsic motivation and attention for learning: The OPTIMAL theory of motor learning. Psychon. Bull. Rev..

[3] Lewthwaite R., Wulf G. (2017). Optimizing motivation and attention for motor performance and learning. Curr. Opin. Psychol..

[4] Magill R.A., Anderson D.I. (2007). Motor Learning and Control: Concepts and Applications.

[5] Wulf G., Hoss M., Prinz W. (1998). Instructions for motor learning: Differential effects of internal versus external focus of attention. J. Mot. Behav..

[6] Marchant D.C., Clough P.J., Crawshaw M. (2007). The effects of attentional focusing strategies on novice dart-throwing performance and their task experiences. Int. J. Sport Exerc. Psychol..

[7] Wulf G., Prinz W. (2001). Directing attention to movement effects enhances learning: A review. Psychon. Bull. Rev..

[8] Wulf G. (2007). Attentional focus and motor learning: A review of 10 years of research. Gabriele Wulf on Attention Focus and Motor Learning. Target Artic. E-J. Beweg. Train..

[9] Wulf G., McNevin N., Shea C.H. (2001). The automaticity of complex motor skill learning as a function of attentional focus. Q. J. Exp. Psychol. A.

[10] Zentgraf K., Lorey B., Bischoff M., Zimmermann K., Stark R., Munzert J. (2009). Neural correlates of attentional focusing during finger movements: A fMRI study. J. Mot. Behav..

[11] Kuhn Y.A., Keller M., Ruffieux J., Taube W. (2017). Adopting an external focus of attention alters intracortical inhibition within the primary motor cortex. Acta Physiol..

[12] Kuhn Y.A., Keller M., Ruffieux J., Taube W. (2017). Intracortical Inhibition Within the Primary Motor Cortex Can Be Modulated by Changing the Focus of Attention. J. Vis. Exp..

[13] Kuhn Y.A., Keller M., Lauber B., Taube W. (2018). Surround inhibition can instantly be modulated by changing the attentional focus. Sci. Rep..

[14] Zachry T., Wulf G., Mercer J., Bezodis N. (2005). Increased movement accuracy and reduced EMG activity as the result of adopting an external focus of attention. Brain Res. Bull..

[15] Vance J., Wulf G., Tollner T., McNevin N., Mercer J. (2004). EMG activity as a function of the performer’s focus of attention. J. Mot. Behav..

[16] Marchant D.C., Greig M., Scott C. (2009). Attentional focusing instructions influence force production and muscular activity during isokinetic elbow flexions. J. Strength Cond. Res..

[17] Wulf G., Dufek J.S., Lozano L., Pettigrew C. (2010). Increased jump height and reduced EMG activity with an external focus. Hum. Mov. Sci..

[18] Snodgrass S.J., Heneghan N.R., Tsao H., Stanwell P.T., Rivett D.A., Van Vliet P.M. (2014). Recognising neuroplasticity in musculoskeletal rehabilitation: A basis for greater collaboration between musculoskeletal and neurological physiotherapists. Man. Ther..

[19] Rossettini G., Cecchetto S., Geri T., Testa M., Zimoli A., Signori A. (2011). Effect of attentional focus instructions on motor learning and performance of patients with central nervous system and musculoskeletal disorders: A systematic review. Ital. J. Physiother..

[20] Sturmberg C., Marquez J., Heneghan N., Snodgrass S., van Vliet P. (2013). Attentional focus of feedback and instructions in the treatment of musculoskeletal dysfunction: A systematic review. Man. Ther..

[21] Park S.H., Yi C.W., Shin J.Y., Ryu Y.U. (2015). Effects of external focus of attention on balance: A short review. J. Phys. Ther. Sci..

[22] Hunt C., Paez A., Folmar E. (2017). The impact of attentional focus on the treatment of musculoskeletal and movement disorders. Int. J. Sports Phys. Ther..

[23] Moher D., Tsertsvadze A., Tricco A.C., Eccles M., Grimshaw J., Sampson M., Barrowman N. (2008). When and how to update systematic reviews. Cochrane Database Syst. Rev..

[24] Garner P., Hopewell S., Chandler J., MacLehose H., Akl E.A., Beyene J., Chang S., Churchill R., Dearness K., Guyatt G. (2016). When and how to update systematic reviews: Consensus and checklist. BMJ.

[25] Liberati A., Altman D.G., Tetzlaff J., Mulrow C., Gotzsche P.C., Ioannidis J.P.A., Clarke M., Devereaux P.J., Kleijnen J., Moher D. (2009). The PRISMA statement for reporting systematic reviews and meta-analyses of studies that evaluate healthcare interventions: Explanation and elaboration. BMJ.

[26] Moher D., Liberati A., Tetzlaff J., Altman D.G. (2009). Preferred reporting items for systematic reviews and meta-analyses: The PRISMA statement. PLoS Med..

[27] Cicchetti D.V., Allison T. (1971). A new procedure for assessing reliability of scoring EEG sleep recordings. Am. J. EEG Technol..

[28] Sherrington C., Herbert R.D., Maher C.G., Moseley A.M. (2000). PEDro. A database of randomized trials and systematic reviews in physiotherapy. Man. Ther..

[29] Verhagen A.P., de Vet H.C., de Bie R.A., Kessels A.G., Boers M., Bouter L.M., Knipschild P.G. (1998). The Delphi list: A criteria list for quality assessment of randomized clinical trials for conducting systematic reviews developed by Delphi consensus. J. Clin. Epidemiol..

[30] De Morton N.A. (2009). The PEDro scale is a valid measure of the methodological quality of clinical trials: A demographic study. Aust. J. Physiother..

[31] Maher C.G., Sherrington C., Herbert R.D., Moseley A.M., Elkins M. (2003). Reliability of the PEDro scale for rating quality of randomized controlled trials. Phys. Ther..

[32] Foley N.C., Bhogal S.K., Teasell R.W., Bureau Y., Speechley M.R. (2006). Estimates of quality and reliability with the physiotherapy evidence-based database scale to assess the methodology of randomized controlled trials of pharmacological and nonpharmacological interventions. Phys. Ther..

[33] Foley N.C., Teasell R.W., Bhogal S.K., Doherty T., Speechley M.R. (2003). The efficacy of stroke rehabilitation: A qualitative review. Top. Stroke Rehabil..

[34] DePaul V., Wishart L., Bramwell A., Hart V., Home K., Pappas L., Pleasance L., Lee T.D. (2009). Impact of focus of attention instructions on walking performance in individuals with and without history of stroke. Physiother. Can..

[35] Johnson L., Burridge J.H., Demain S.H. (2013). Internal and external focus of attention during gait re-education: An observational study of physical therapist practice in stroke rehabilitation. Phys. Ther..

[36] Kal E., van den Brink H., Houdijk H., van der Kamp J., Goossens P.H., van Bennekom C., Scherder E. (2017). How physical therapists instruct patients with stroke: An observational study on attentional focus during gait rehabilitation after stroke. Disabil. Rehabil..

[37] McAlister R. (2006). The Effects of Attentional Focus Instructions on Simulated Upper Extremity Amputees’ Movement Kinematics When Learning a Novel Functional Task. Ph.D. Thesis.

[38] Silsupadol P., Shumway-Cook A., Lugade V., van Donkelaar P., Chou L.-S., Mayr U., Woollacott M.H. (2009). Effects of single-task versus dual-task training on balance performance in older adults: A double-blind, randomized controlled trial. Arch. Phys. Med. Rehabil..

[39] Verrel J., Lovden M., Schellenbach M., Schaefer S., Lindenberger U. (2009). Interacting effects of cognitive load and adult age on the regularity of whole-body motion during treadmill walking. Psychol. Aging.

[40] Shafizadeh M., Platt G.K., Mohammadi B. (2013). Effects of different focus of attention rehabilitative training on gait performance in Multiple Sclerosis patients. J. Bodyw. Mov. Ther..

[41] Gray R. (2015). Differences in Attentional Focus Associated with Recovery from Sports Injury: Does Injury Induce an Internal Focus?. J. Sport Exerc. Psychol..

[42] Landers M.R., Hatlevig R.M., Davis A.D., Richards A.R., Rosenlof L.E. (2016). Does attentional focus during balance training in people with Parkinson’s disease affect outcome? A randomised controlled clinical trial. Clin. Rehabil..

[43] Cirstea M.C., Levin M.F. (2007). Improvement of arm movement patterns and endpoint control depends on type of feedback during practice in stroke survivors. Neurorehabil. Neural Repair.

[44] Durham K., Van Vliet P.M., Badger F., Sackley C. (2009). Use of information feedback and attentional focus of feedback in treating the person with a hemiplegic arm. Physiother. Res. Int..

[45] Fasoli S.E., Trombly C.A., Tickle-Degnen L., Verfaellie M.H. (2002). Effect of instructions on functional reach in persons with and without cerebrovascular accident. Am. J. Occup. Ther..

[46] Landers M., Wulf G., Wallmann H., Guadagnoli M. (2005). An external focus of attention attenuates balance impairment in patients with Parkinson’s disease who have a fall history. Physiotherapy.

[47] Wulf G., Landers M., Lewthwaite R., Töllner T. (2009). External focus instructions reduce postural instability in individuals with Parkinson disease. Phys. Ther..

[48] Kakar C., Zia N., Sehgal S., Khushwaha S. (2013). Effect of external and internal focus of attention on acquisition, retention, and transfer phase of motor learning in Parkinson’s disease. Hong Kong Physiother. J..

[49] Durham K.F., Sackley C.M., Wright C.C., Wing A.M., Edwards M.G., van Vliet P. (2014). Attentional focus of feedback for improving performance of reach-to-grasp after stroke: A randomised crossover study. Physiotherapy.

[50] Mückel S., Mehrholz J. (2014). Immediate effects of two attention strategies on trunk control on patients after stroke. A randomized controlled pilot trial. Clin. Rehabil..

[51] Kal E.C., van der Kamp J., Houdijk H., Groet E., van Bennekom C.A.M., Scherder E.J.A. (2015). Stay Focused! The Effects of Internal and External Focus of Attention on Movement Automaticity in Patients with Stroke. PLoS ONE.

[52] Beck E.N., Almeida Q.J. (2017). Dopa-Responsive Balance Changes Depend on Use of Internal Versus External Attentional Focus in Parkinson Disease. Phys. Ther..

[53] Sakurada T., Nakajima T., Morita M., Hirai M., Watanabe E. (2017). Improved motor performance in patients with acute stroke using the optimal individual attentional strategy. Sci. Rep..

[54] Kim G.J., Hinojosa J., Rao A.K., Batavia M., O’Dell M.W. (2017). A Randomized Trial on the Effects of Attentional Focus on Motor Training of the Upper Extremity Using Robotics with Individuals after Chronic Stroke. Arch. Phys. Med. Rehabil..

[55] Laufer Y., Rotem-Lehrer N., Ronen Z., Khayutin G., Rozenberg I. (2007). Effect of attention focus on acquisition and retention of postural control following ankle sprain. Arch. Phys. Med. Rehabil..

[56] Rotem-Lehrer N., Laufer Y. (2007). Effect of focus of attention on transfer of a postural control task following an ankle sprain. J. Orthop. Sports Phys. Ther..

[57] Gokeler A., Benjaminse A., Welling W., Alferink M., Eppinga P., Otten B. (2015). The effects of attentional focus on jump performance and knee joint kinematics in patients after ACL reconstruction. Phys. Ther. Sport.

[58] Fisk J.D., Goodale M.A. (1988). The effects of unilateral brain damage on visually guided reaching: Hemispheric differences in the nature of the deficit. Exp. brain Res..

[59] Boyd L.A., Winstein C.J. (2001). Implicit motor-sequence learning in humans following unilateral stroke: The impact of practice and explicit knowledge. Neurosci. Lett..

[60] Peh S.Y.-C., Chow J.Y., Davids K. (2011). Focus of attention and its impact on movement behaviour. J. Sci. Med. Sport.

[61] Green T.D., Flowers J.H. (1991). Implicit versus Explicit Learning Processes in a Probabilistic, Continuous Fine-Motor Catching Task. J. Mot. Behav..

[62] Mehdizadeh M., Lajevardi L., Habibi S.A.H., ArabBaniasad M., Baghoori D., Daneshjoo F., Taghizadeh G. (2016). The association between fear of falling and quality of life for balance impairments based on hip and ankle strategies in the drug On- and Off-phase of patients with Idiopathic Parkinson’ disease. Med. J. Islam. Repub. Iran.

[63] Smania N., Corato E., Tinazzi M., Stanzani C., Fiaschi A., Girardi P., Gandolfi M. (2010). Effect of balance training on postural instability in patients with Idiopathic Parkinson’s disease. Neurorehabil. Neural Repair.

[64] Wulf G., Tollner T., Shea C.H. (2007). Attentional focus effects as a function of task difficulty. Res. Q. Exerc. Sport.

[65] Funkiewiez A., Ardouin C., Cools R., Krack P., Fraix V., Batir A., Chabardes S., Benabid A.-L., Robbins T.W., Pollak P. (2006). Effects of levodopa and subthalamic nucleus stimulation on cognitive and affective functioning in Parkinson’s disease. Mov. Disord..

[66] Maricle R.A., Nutt J.G., Valentine R.J., Carter J.H. (1995). Dose-response relationship of levodopa with mood and anxiety in fluctuating Parkinson’s disease: A double-blind, placebo-controlled study. Neurology.

[67] Jazaeri S.Z., Azad A., Mehdizadeh H., Habibi S.A., Mandehgary Najafabadi M., Saberi Z.S., Rahimzadegan H., Moradi S., Behzadipour S., Parnianpour M. (2018). The effects of anxiety and external attentional focus on postural control in patients with Parkinson’s disease. PLoS ONE.

[68] Holme E., Magnusson S.P., Becher K., Bieler T., Aagaard P., Kjaer M. (1999). The effect of supervised rehabilitation on strength, postural sway, position sense and re-injury risk after acute ankle ligament sprain. Scand. J. Med. Sci. Sports.

[69] Blackburn J.T., Padua D.A. (2008). Influence of trunk flexion on hip and knee joint kinematics during a controlled drop landing. Clin. Biomech..

[70] Hewett T.E., Myer G.D., Ford K.R., Paterno M.V., Quatman C.E. (2012). The 2012 ABJS Nicolas Andry Award: The Sequence of Prevention: A Systematic Approach to Prevent Anterior Cruciate Ligament Injury. Clin. Orthop. Relat. Res..

[71] Tsai L.-C., Powers C.M. (2012). Increased Hip and Knee Flexion during Landing Decreases Tibiofemoral Compressive Forces in Women Who Have Undergone Anterior Cruciate Ligament Reconstruction. Am. J. Sports Med..

[72] Benjaminse A., Gokeler A., Dowling A.V., Faigenbaum A., Ford K.R., Hewett T.E., Onate J.A., Otten B., Myer G.D. (2015). Optimization of the anterior cruciate ligament injury prevention paradigm: Novel feedback techniques to enhance motor learning and reduce injury risk. J. Orthop. Sports Phys. Ther..

[73] Rossettini G., Testa M., Vicentini M., Manganotti P. (2017). The Effect of Different Attentional Focus Instructions during Finger Movement Tasks in Healthy Subjects: An Exploratory Study. Biomed Res. Int..

[74] Wulf G., Shea C., Lewthwaite R. (2010). Motor skill learning and performance: A review of influential factors. Med. Educ..

[75] Dwan K., Gamble C., Williamson P.R., Kirkham J.J. (2013). Systematic Review of the Empirical Evidence of Study Publication Bias and Outcome Reporting Bias—An Updated Review. PLoS ONE.

[76] Song F., Parekh S., Hooper L., Loke Y.K., Ryder J., Sutton A.J., Hing C., Kwok C.S., Pang C., Harvey I. (2010). Dissemination and publication of research findings: An updated review of related biases. Health Technol. Assess..

